# Rare Causes of Musculoskeletal Pain: Thinking beyond Common Rheumatologic Diseases

**DOI:** 10.1155/2024/6540026

**Published:** 2024-01-18

**Authors:** Julia F. Charles, Alan O. Malabanan, Stan Krolczyk, Kathryn M. Dahir

**Affiliations:** ^1^Brigham and Women's Hospital, Harvard Medical School, Boston, MA, USA; ^2^Boston Medical Center, Boston, MA, USA; ^3^Ultragenyx Pharmaceutical Inc., Novato, CA, USA; ^4^Vanderbilt University Medical Center, Nashville, TN, USA

## Abstract

**Objectives:**

Rare metabolic bone diseases can present with symptoms mimicking more common rheumatological conditions including spondyloarthritis, osteoarthritis, and fibromyalgia. Increasing awareness of these rare diseases within the rheumatology community is vital to ensure that affected patients are diagnosed and appropriately treated. The literature includes several reports of tumour-induced osteomalacia initially diagnosed as rheumatic disease, but other rare diseases such as X-linked hypophosphatemia (XLH) and hypophosphatasia (HPP) also deserve attention. Here, we describe two cases of adult patients incorrectly diagnosed with ankylosing spondylitis and osteoarthritis who, upon referral to a metabolic bone disease specialist, were subsequently diagnosed with XLH and HPP, respectively, profoundly altering their management.

**Methods:**

The cases were collected from Brigham and Women's Hospital, Boston, MA, USA, and Vanderbilt University Medical Center, Nashville, TN, USA.

**Results:**

Details of the patients' respective medical and family histories are presented, and the clinical and biochemical investigations undertaken to reach the correct diagnoses are described.

**Conclusion:**

Rheumatologists should be encouraged to think beyond common rheumatological diseases when faced with symptoms such as bone pain, muscle pain, and stiffness, especially when accompanied by manifestations including atraumatic fractures, poor dentition, and hearing loss. In cases where one of these rare diseases is suspected, referral to a metabolic bone disease specialist for confirmation of diagnosis is encouraged as effective treatment options have recently become available.

## 1. Introduction

Patients with bone, joint and/or muscle pain, and stiffness are frequently referred to rheumatologists for diagnosis and management. The differential for these complaints is long, and rheumatologists generally take pride in their diagnostic acumen and broad knowledge of medicine. While in most cases, these symptoms lead to a common rheumatological diagnosis, whether it may be osteoarthritis, fibromyalgia, or an inflammatory arthritis, several rare metabolic bone diseases (MBDs) can mimic the presentation of these more common diagnoses, as highlighted by the cases presented below.

Disorders of phosphate wasting, such as tumour-induced osteomalacia (TIO), may be misdiagnosed as fibromyalgia, polymyalgia rheumatica, psoriatic arthritis (PsA), and ankylosing spondylitis (AS) [1–3]. Other rare MBDs with rheumatological-like symptoms that have received less attention include two rare genetic disorders, X-linked hypophosphatemia (XLH) and hypophosphatasia (HPP). XLH is due to inactivating mutations in phosphate-regulating endopeptidase homolog X-linked (*PHEX*), leading to chronic phosphate wasting, skeletal and dental defects in childhood, and chronic musculoskeletal morbidities in adulthood [4]. HPP results from mutations in the *ALPL gene*, encoding tissue-nonspecific alkaline phosphatase, and is characterized by reduced activity of tissue-nonspecific alkaline phosphatase (ALP) and impaired mineralization of bones and teeth, as well as musculoskeletal pain, fatigue, and other systemic symptoms [5].

In this concise report, we describe two cases of adult patients incorrectly diagnosed with rheumatological conditions who, upon referral to an MBD specialist, were diagnosed with XLH and HPP, respectively. We outline the clinical and biochemical investigations undertaken to reach these diagnoses and highlight the pitfalls that may be encountered in the clinical workup of such cases, encouraging rheumatologists to think beyond common causes when presented with symptoms of bone and muscle pain.

## 2. Methods

The cases were collected from Brigham and Women's Hospital (BWH), Boston, MA, USA, and Vanderbilt University Medical Center (VUMC), Nashville, TN, USA. Written informed consent was obtained from both patients. Data were extracted from electronic medical records, including details of clinical features; medical and family history; physical, biochemical, and imaging assessments; and treatment. Our study complies with the Declaration of Helsinki and Health Insurance Portability and Accountability Act (HIPAA) regulations.

## 3. Case Presentation

### 3.1. Case Report 1

**Figure 1:**
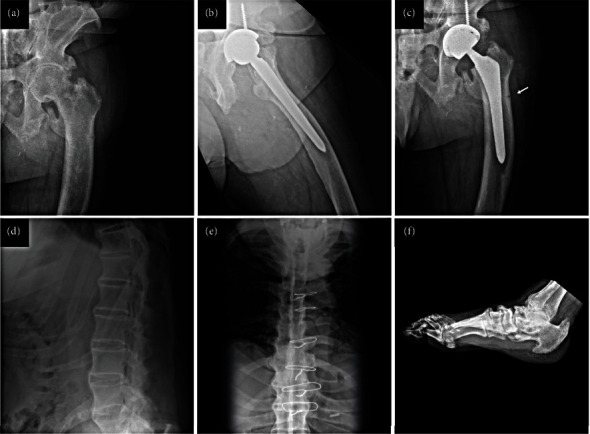
The X-ray images from our XLH case (a–c). Preoperative films of the left hip (a) demonstrate prominent enthesophytes of the left iliac wing and trochanters, initially misdiagnosed as sequelae of AS. The femoral bowing seen would be atypical of AS. The patient had a successful total hip arthroplasty of the left hip (b), which was later complicated by pseudofracture ((c), arrow) characteristic of XLH but not AS. To further illustrate how XLH can mimic spondyloarthritis on imaging, lateral (d) and anterior posterior (e) thoracic spine films demonstrate flowing syndesmophytes and a lateral ankle X-ray scan shows an Achilles enthesophyte (f).

A 64-year-old male presented to VUMC in 2019 following his daughter's XLH diagnosis. He had been diagnosed with AS at age 29 years based on progressive back pain and stiffness, hip pain and joint space narrowing, and a history of ankle surgery for enthesopathy at age 19 years. His imaging was notable for diffuse enthesophytes of the spine and Achilles (Figure 1), interspinous ligament calcification, and bilateral sacroiliac joint fusion, described as “severe AS” by both his rheumatologist and orthopaedic surgeon, as well as bilateral hip arthroplasties. At presentation, he was using hydrocodone to treat his pain, and he had not experienced symptomatic improvement with indomethacin or other nonsteroidal anti-inflammatory drugs and had previously tried and failed therapy with adalimumab. Historical features atypical for AS included bilateral bowing of his proximal femoral shafts with subtrochanteric lateral proximal insufficiency fractures (on X-ray) and progressive hearing loss since the age of 9 years. A review of 20 years of dental records also revealed a history of poor dentition, including multiple abscesses (in numbers 3, 8, 14, 15, and 19), dental fractures, extractions (of 14, 26, and 31), and antibiotics for dental infections on an almost yearly basis. Pertinent laboratory values included (normal limits in brackets) inorganic phosphate 1.9 (2.5–4.5) mg/dL, ALP 148 (40–129) U/L, serum creatinine 0.74 (0.7–1.3) mg/dL, spot urine creatinine 76 (20–320) mg/dL, parathyroid hormone (PTH) 115 (16–77) pg/mL, 1,25(OH)_2_ vitamin D 49.9 (19.7–79.3) pg/mL, and serum calcium 9.8 (8.4–10.5) mg/dL. The 24-hour urine phosphate of 1300 mg (226–1797 mg) was inappropriately normal, consistent with renal phosphate wasting. Testing for HLA-B27 was negative. Genetic testing revealed two mutations in *PHEX* both classified as likely pathogenic (transcript NM_000444.5; c.*∗*231A > G; duplication exon 13–15) [6], resulting in a diagnosis of XLH. This PHEX variant has emerged as a common cause of XLH in North America as described in a recent report of a five-generation American kindred of 22 individuals with XLH, including the case presented here [7]. Treatment with burosumab (1 mg/kg every 4 weeks), an inhibitor of fibroblast growth factor 23 (FGF23) [8], was initiated with improvements in laboratory values (inorganic phosphate 2.4 mg/dL, ALP 142 U/L), timed up and go assessment (12.95 seconds at year 1 and 12.35 at year 3 from a baseline of 13.86 seconds), and patient-reported outcomes. Patient-reported outcomes included the WOMAC pain score (0–20 with 0 being least) decreased from a baseline of 7 to 2 at year 1 and 4 at year 3, WOMAC stiffness (0–8 with 0 being least) decreased from a baseline of 5 to 3 at year 1 and 4 at year 3, WOMAC activities of daily living (0–68 with 0 being least) decreased from a baseline of 37 to 30 at year 1 and 33 at year 3, and a PROMIS physical function T score of 36.5 at year 1 and 35.8 at year 3 from a baseline of 30.4, with no new fractures.

One of the patient's brothers was subsequently referred for XLH assessment. Similar to our patient, he had a history of stiffness and hip/back pain, as well as heel enthesopathies and hip osteonecrosis, and was diagnosed with AS aged 56 years by his rheumatologist. He had not responded to meloxicam or adalimumab. Notably, he also had a history of poor dentition and progressive hearing loss. Genetic testing subsequently confirmed a diagnosis of XLH. A third brother with an AS diagnosis has also recently been diagnosed with XLH.

### 3.2. Case Report 2

**Figure 2:**
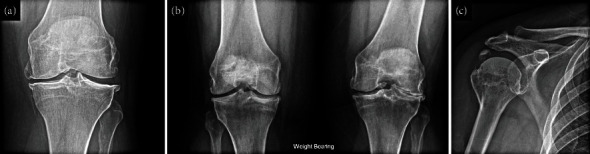
X-ray images from our HPP case, illustrating calcium pyrophosphate dihydrate deposition in the left knee (a), progressing to severe lateral joint space narrowing (b). Our patient also had calcific tendonitis in the right shoulder (c).

A 57-year-old woman was referred for osteoporosis evaluation at BWH after a fragility fracture of the humerus. She had a history of fractures, including right calcaneal fracture, clinically diagnosed metatarsal stress fracture, bilateral patellar fractures requiring surgery, and a displaced comminuted shoulder fracture. She had a longstanding complaint of diffuse musculoskeletal pain and fatigue, for which she had been evaluated by a rheumatologist and noted to have calcium pyrophosphate dihydrate deposition (CPPD) of the scapholunate ligament and both knees with severe left lateral joint space narrowing and calcific tendonitis in her shoulder (Figure 2). An evaluation for haemochromatosis as a cause of her CPPD was negative. She was given a diagnosis of CPPD and osteoarthritis as a cause of her musculoskeletal symptoms and underwent a total knee arthroplasty. Additional history obtained during her evaluation for metabolic bone disease included a history of hip dysplasia, loss of deciduous teeth at age 4 years, and multiple cavities. She had no family history of bone disease, but her father was edentulous by age 30 years. Dual-energy X-ray absorptiometry indicated normal bone density (lowest T score −0.7 at the femoral neck). A review of laboratory values revealed that ALP had been below 25 U/L for the past 10 years. Pertinent laboratory values (normal limits in brackets) included an ALP of 22 (40–129) IU/L, with the remainder of the complete metabolic panel being normal, and a vitamin B6 level of 277 (5–50) *μ*g/L. The remainder of her evaluation for secondary causes of bone fragility was also normal, including 25-hydroxyvitamin D 49 (30–50) ng/mL, calcium 10.3 (8.6–10.3) mg/dL, inorganic phosphate 4.2 (2.5–4.5) mg/dL, and PTH 24 (14–65) pg/mL.

The patient was referred to endocrine genetics because of suspicion of HPP; genetic testing revealed a heterozygous likely pathogenic mutation in the *ALPL* gene (transcript NM_000478.5; c.303C > A [p.Tyr101*∗*] resulting in a premature stop codon). This mutation has previously been reported in a patient with adult-onset osteoporosis and low ALP [9]. Random urine assessment of amino acids by liquid chromatography with tandem mass spectrometry indicated elevated levels of phosphoethanolamine (65 nmol/mg Cr with normal limit: <48). An analysis of bone turnover markers indicated low/normal bone turnover: CTX 176 (normal: 104–1008) pg/mL, P1NP 29 (16–96) *μ*g/L, and osteocalcin 10 (9–42) ng/mL. A skeletal survey of the axial and proximal appendicular skeleton did not reveal additional fractures/pseudofractures; although bone scintigraphy is a more sensitive imaging approach to detect pseudofracture, it was not performed in this case. Following her HPP diagnosis, enzyme replacement therapy (asfotase alfa 80 mg subcutaneously 6 days/week) led to improvements in repeat chair stand assessment (28 seconds at baseline, 16 seconds approximately 1 month after starting treatment, stabilizing at 13 seconds after 6 months) and subjective improvement in fatigue but no change in joint pain.

## 4. Discussion

Our cases highlight the importance of differential diagnosis of rare MBDs versus more common rheumatological diseases, adding to the previously published cases highlighting misdiagnosis of XLH and HPP as AS, osteoporosis, PsA, and fibromyalgia [10–13].

As common symptoms of XLH and HPP may mimic symptoms associated with rheumatological diseases, the workup of suspected rare MBDs should involve taking a thorough patient history and full physical examination. As identified in our cases, a history of atraumatic fractures should trigger suspicion of disorders such as XLH and HPP. A more thorough biochemical assessment, particularly regarding low serum phosphate and alkaline phosphatase levels, could have raised suspicion of XLH and HPP, respectively, earlier in both of our cases. While ALP remains part of routine blood chemistry assessment panels in most institutions, the omission of phosphate explains why hypophosphatemia often goes undetected, and is a potential factor contributing to delayed diagnoses.

Upon referral to VUMC, to our knowledge, phosphate levels had never previously been checked in our patient. Further investigations showed that inorganic phosphate was below normal. Several recent detailed reviews of the diagnostic approach to hypophosphatemic disorders, including XLH, have been published [14, 15]. In general, an unexplained serum phosphate level below the normal reference range (which may vary by age and/or sex) should raise suspicion of hypophosphatemia and prompt measurement of renal tubular reabsorption of phosphate (TmP/GFR) or fractional excretion of phosphate or 24-hour urine phosphorous and calcium levels to differentiate between malabsorption and phosphate wasting [16, 17]. Assessment of phosphate wasting in urine is an inexpensive and standard test for investigating and interpreting hypophosphatemia, the results of which guide further clinical and biochemical investigations.

Our patient's 1,25(OH)_2_ vitamin D was inappropriately within the normal range, PTH and ALP levels were elevated, and there was evidence of phosphate wasting (inappropriately normal 24 hour urine phosphate in the face of serum hypophosphataemia), all consistent with XLH. While, in the absence of a clear family history, it is recommended practice to measure TmP/GFR and serum FGF23 levels when XLH is suspected [14, 15], these tests were not conducted in our case due to a positive family history of XLH and the evidence of phosphate wasting based on 24-hour urine phosphorus results. The patient's daughter had recently received a diagnosis of XLH, so we elected to expedite diagnosis by genetic testing.

While at the time of genetic diagnosis, our patient's exuberant enthesophytes would have been difficult to confuse with those of AS (Figures 1(a)–1(c)), X-ray findings in early XLH can be similar to those of AS, which can make differential diagnosis more challenging (Figures 1(d)–1(f)). The fact that our XLH case was White and HLA-B27 negative is atypical of AS, in people of European ancestry, approximately 90% of AS cases are HLA-B27 positive [18]. The lack of response to anti-inflammatory treatment was also atypical. All these clinical clues were missed and not acted upon by the initial physicians who managed the patient.

In our XLH case, a history of bilateral hip osteonecrosis and femoral head fractures is atypical of AS. The type of fracture observed can also help to distinguish between XLH (predominantly medial cortex pseudofractures) and HPP (lateral cortex pseudofractures) [16, 19]. In our case, pseudofractures were detectable on plain films; however, in more subtle cases where XLH is suspected, a radionuclide bone scan can be helpful to detect pseudofractures. With respect to phosphate-wasting diseases, the pain, stiffness, kyphosis, and progressive deafness observed in our XLH case are common manifestations of this disease [4]. Furthermore, a history of childhood rickets and leg bowing are suggestive of XLH in particular because of its lifelong nature, and this may help in the differential diagnosis versus TIO, which presents with a more abrupt time course [2, 4, 20]. In addition to AS, our XLH case's rheumatologist also considered a diagnosis of diffuse idiopathic skeletal hyperostosis (DISH), due to ectopic bone formation in the spine. However, in our experience, DISH affects older individuals, ligamentous calcification typically is limited to the thoracic spine, and enthesophytes are asymmetric. Spinal stenosis due to extensive spinal ligament calcification, as seen in our patient, is common in XLH and can be evaluated with a whole-spine MRI scan, although this has not yet been performed in his case.

HPP is characterized by below normal levels of ALP. Diagnostic criteria for HPP in adults have recently been published (PMID 37982856) [21]. Notably, low ALP is not pathognomonic, and other conditions such as eating disorders, thyroid dysfunction, milk-alkali syndrome, multiple myeloma, celiac disease, and zinc deficiency should be ruled out as part of the diagnostic workup; elevated levels of vitamin B6 concomitant with low ALP levels can be used to differentiate HPP from other conditions. Serum vitamin D and PTH levels are normal with normal or elevated calcium and phosphate levels. Elevated levels of urinary phosphoethanolamine may also be observed [21, 22]. Diagnosis is usually confirmed by genetic analysis of the *ALPL* gene. In our case, ALP had been persistently below 25 U/L for the past 10 years and was 22 U/L at diagnosis. This crucial abnormality had not been investigated further until the patient was referred for specialist assessment at BWH.

Patients with HPP can present with a broad spectrum of symptoms. A detailed discussion of HPP presentation is outside the scope of this article (reviewed in reference 5), and indeed, rheumatologists are only likely to encounter patients with mild HPP. CPPD disease, as a result of a high ratio of inorganic pyrophosphate to phosphate ions, is among the characteristic traits of HPP observed in our case that rheumatologists should be cognizant of [5, 23]. CPPD disease was present in both the knees and scapholunate ligament of our case; other commonly affected sites in patients with HPP include the triangular fibrocartilage of the wrist and less commonly the hips, shoulders, elbows, ankles, and hands [5, 23].

Our XLH and HPP cases also presented with poor dentition, including evidence of abscesses, extractions, and dental fractures because of low phosphate and impaired mineralization, respectively. Subtle differences in the dental manifestations of HPP and XLH may be evident, with the former more often associated with losing primary dentition with the root intact due to deficient cementum and the latter with spontaneous dental abscess and delayed tooth eruption [24].

While assessing family history is an important step in diagnosing XLH and HPP, both of which are usually hereditary diseases, *de novo* genetic mutations should also be considered in both diseases. As highlighted in our cases, a positive family history of atraumatic fractures and poor dentition can be suggestive of XLH or HPP. A confirmed diagnosis of XLH or HPP should trigger assessment of close relatives, as occurred in our XLH case who was referred following diagnosis of XLH in his daughter. Family screening was not performed in our HPP case as the patient's parents were deceased at the time of diagnosis and her offspring declined screening. Genetic testing is recommended to confirm the presence of pathologic mutations in the *PHEX* or *ALPL* genes, and this also offers a definitive way of differentiating XLH from TIO.

## 5. Conclusions

Rheumatologists should consider rare MBDs when faced with signs and symptoms mimicking more common rheumatological conditions. Appropriate clinical and biochemical investigations are required to aid differential diagnosis and avoid the delays in treatment, gradual worsening of symptoms, frequent fractures, loss of mobility, and diminished quality of life observed in our cases. When one of these rare diseases is suspected, referral to an MBD specialist for confirmation of diagnosis is encouraged as effective treatment options are available.

## Data Availability

The data underlying this article are available in the article.

## References

[1] Jan de Beur S. M., Minisola S., Xia W.-B. (2023). Global guidance for the recognition, diagnosis, and management of tumor-induced osteomalacia. *Journal of Internal Medicine*.

[2] Florenzano P., Hartley I. R., Jimenez M., Roszko K., Gafni R. I., Collins M. T. (2021). Tumor-induced osteomalacia. *Calcified Tissue International*.

[3] Feng J., Jiang Y., Wang O. (2017). The diagnostic dilemma of tumor induced osteomalacia: a retrospective analysis of 144 cases. *Endocrine Journal*.

[4] Dahir K., Roberts M. S., Krolczyk S., Simmons J. H. (2020). X-linked hypophosphatemia: a new era in management. *Journal of the Endocrine Society*.

[5] Fenn J. S., Lorde N., Ward J. M., Borovickova I. (2021). Hypophosphatasia. *Journal of Clinical Pathology*.

[6] Rush E. T., Johnson B., Aradhya S. (2022). Molecular diagnoses of X-linked and other genetic hypophosphatemias: results from a sponsored genetic testing Program. *Journal of Bone and Mineral Research*.

[7] Dahir K. M., Black M., Gottesman G. S. (2022). X-linked hypophosphatemia caused by the prevailing North American *PHEX* variant c.∗231A>G; exon 13-15 duplication is often misdiagnosed as ankylosing spondylitis and manifests in both men and women. *Journal of Bone and Mineral Research Plus*.

[8] Lamb Y. N. (2018). Burosumab: first global approval. *Drugs*.

[9] Alonso N., Larraz-Prieto B., Berg K. (2020). Loss-of-Function mutations in the ALPL gene presenting with adult onset osteoporosis and low serum concentrations of total alkaline phosphatase. *Journal of Bone and Mineral Research*.

[10] Takase R., Nakano Y., Hasegawa K., Otsuka F. (2020). X-Linked hypophosphatemia (XLH) mimicking rheumatic disease. *Internal Medicine*.

[11] Ozbayrak S. S. (2020). Previously undiagnosed hypophosphatemic rickets presenting like ankylosing spondylitis in adulthood: a case report. *Therapeutic Clinic*.

[12] Rodziewicz M. S., Moss K. (2018). 79. Hypophosphatasia presenting as seronegative arthritis and mistaken for psoriatic arthritis. *Rheumatology Advances in Practice*.

[13] García-Fontana C., Villa-Suárez J. M., Andújar-Vera F. (2019). Epidemiological, clinical and genetic study of hypophosphatasia in A Spanish population: identification of two novel mutations in the alpl gene. *Scientific Reports*.

[14] Tebben P. J. (2022). Hypophosphatemia: a practical guide to evaluation and management. *Endocrine Practice*.

[15] Florenzano P., Cipriani C., Roszko K. L. (2020). Approach to patients with hypophosphataemia. *Lancet Diabetes and Endocrinology*.

[16] Haffner D., Emma F., Eastwood D. M. (2019). Clinical practice recommendations for the diagnosis and management of X-linked hypophosphataemia. *Nature Reviews Nephrology*.

[17] Aljuraibah F., Bacchetta J., Brandi M. L. (2022). An expert perspective on phosphate dysregulation with a focus on chronic hypophosphatemia. *Journal of Bone and Mineral Research*.

[18] Reveille J. D. (2006). Major histocompatibility genes and ankylosing spondylitis. *Best Practice & Research Clinical Rheumatology*.

[19] Genest F., Claußen L., Rak D., Seefried L. (2021). Bone mineral density and fracture risk in adult patients with hypophosphatasia. *Osteoporosis International*.

[20] Colazo J. M., DeCorte J. A., Gillaspie E. A., Folpe A. L., Dahir K. M. (2021). Hiding in plain sight: gene panel and genetic markers reveal 26-year undiagnosed tumor-induced osteomalacia of the rib concurrently misdiagnosed as X-linked hypophosphatemia. *BoneKEy Reports*.

[21] Brandi M. L., Khan A. A., Rush E. T. (2023). The challenge of hypophosphatasia diagnosis in adults: results from the HPP International Working Group Literature Surveillance. *Osteoporosis International*.

[22] Feurstein J., Behanova M., Haschka J. (2022). Identifying adult hypophosphatasia in the rheumatology unit. *Orphanet Journal of Rare Diseases*.

[23] Rosenthal A. K., Ryan L. M. (2016). Calcium pyrophosphate deposition disease. *New England Journal of Medicine*.

[24] Foster B. L., Ramnitz M. S., Gafni R. I. (2014). Rare bone diseases and their dental, oral, and craniofacial manifestations. *Journal of Dental Research*.

